# Immunogenicity and Safety According to Immunosuppressive Drugs and Different COVID-19 Vaccine Platforms in Immune-Mediated Disease: Data from SAFER Cohort

**DOI:** 10.3390/vaccines12121367

**Published:** 2024-12-03

**Authors:** Ketty Lysie Libardi Lira Machado, Ana Paula Neves Burian, Olindo Assis Martins-Filho, José Geraldo Mill, Lunara Baptista Ferreira, Karina Rosemarie Lallemand Tapia, Anna Carolina Simões Moulin, Isac Ribeiro Moulaz, Priscila Dias Cardoso Ribeiro, Vanessa de Oliveira Magalhães, Erika Biegelmeyer, Flávia Maria Matos Melo Campos Peixoto, Sandra Lúcia Euzébio Ribeiro, Camila Maria Paiva França Telles, Juliana Bühring, Natalia Sarzi Sartorio, Vanessa Hax, Rodrigo Poubel Vieira de Rezende, Katia Lino Baptista, Ana Karla Guedes de Melo, Vitor Alves Cruz, Rejane Maria Rodrigues de Abreu Vieira, Renata Henriques de Azevedo, Valderilio Feijó Azevedo, Marcelo de Medeiros Pinheiro, Odirlei André Monticielo, Edgard Torres Dos Reis Neto, Andréa Teixeira-Carvalho, Ricardo Machado Xavier, Emilia Inoue Sato, Viviane Angelina de Souza, Gilda Aparecida Ferreira, Gecilmara Salviato Pileggi, Valeria Valim

**Affiliations:** 1 Hospital Universitário Cassiano Antônio Moraes da Universidade Federal do Espírito Santo (HUCAM-UFES/EBSERH), Vitória 29041-295, ES, Brazil; drakettymachado@gmail.com (K.L.L.L.M.); josegmill@gmail.com (J.G.M.); lunarabferreira70@gmail.com (L.B.F.); karinalallemand@gmail.com (K.R.L.T.); annasmoulin@gmail.com (A.C.S.M.); isacmoulaz@gmail.com (I.R.M.); 2 Centro de Referências para Imunobiológicos Especiais (CRIE) da Secretaria de Saúde do Estado do Espírito Santo, Vitória 29047-105, ES, Brazil; anapaulaburian@gmail.com; 3 Grupo Integrado de Pesquisas em Biomarcadores, Instituto René Rachou, Fundação Oswaldo Cruz (FIOCRUZ-Minas), Belo Horizonte 30130-100, MG, Brazil; oamfilho@gmail.com (O.A.M.-F.); andrea.teixeira@fiocruz.br (A.T.-C.); 4 Escola Paulista de Medicina (EPM), Universidade Federal de São Paulo (UNIFESP), São Paulo 04023-062, SP, Brazil; pri.dcr@gmail.com (P.D.C.R.); vanessa.reumato@gmail.com (V.d.O.M.); erika.biegel@gmail.com (E.B.); flaviacampospeixoto@gmail.com (F.M.M.M.C.P.); mpinheiro@uol.com.br (M.d.M.P.); edgard.torres@unifesp.br (E.T.D.R.N.); eisato@unifesp.br (E.I.S.); gecilmara@gmail.com (G.S.P.); 5 Escola de Medicina, Universidade Federal do Amazonas (UFAM), Manaus 69067-005, AM, Brazil; sandraler04@gmail.com (S.L.E.R.); camilapaiva2003@hotmail.com (C.M.P.F.T.); jubuhring@hotmail.com (J.B.); 6 Hospital de Clínicas de Porto Alegre, Universidade Federal do Rio Grande do Sul (UFRGS), Porto Alegre 90010-150, RS, Brazil; nsartori@hcpa.edu.br (N.S.S.); vhax@hcpa.edu.br (V.H.); omonticielo@gmail.com (O.A.M.); rxavier10@gmail.com (R.M.X.); 7 Universidade Federal Fluminense, Rheumatology, Niteroi 24020-140, RJ, Brazil; ropoubel@id.uff.br (R.P.V.d.R.); linokatia@gmail.com (K.L.B.); 8 Hospital Universitário Lauro Wanderley, Universidade Federal da Paraíba (UFPB), João Pessoa 58051-900, PB, Brazil; anakarlagmelo@gmail.com; 9 Faculdade de Medicina, Universidade Federal de Goiás (UFG), Goiânia 74690-900, GO, Brazil; vitorcruz@ufg.br; 10 Hospital Geral de Fortaleza (HGF), Universidade de Fortaleza (UNIFOR), Fortaleza 60150-160, CE, Brazil; rejaneavieira@gmail.com; 11 Faculdade de Medicina, Universidade Federal de Juiz de Fora, Juiz de Fora 36036-900, MG, Brazil; renata.reumato@gmail.com (R.H.d.A.); vivi.reumato@gmail.com (V.A.d.S.); 12 Edumed Educação em Saúde, Rheumatology, Curitiba 80440-210, PR, Brazil; valderilio@hotmail.com; 13 Locomotor System Department, Faculdade de Medicina, Universidade Federal de Minas Gerais (UFMG), Belo Horizonte 31270-901, MG, Brazil; gildaferreira9@gmail.com

**Keywords:** registries, COVID-19, vaccine, autoimmune disorders, humoral immunity

## Abstract

Background/Objectives: The effectiveness of COVID-19 vaccine in patients with immune-mediated inflammatory diseases (IMID) depends on the underlying disease, immunosuppression degree and the vaccine regimens. We evaluate the safety and immunogenicity of different COVID-19 vaccine schedules. Methods: The SAFER study: “Safety and effectiveness of the COVID-19 Vaccine in Rheumatic Disease”, is a Brazilian multicentric prospective observational phase IV study in the real-life. Data were analyzed after 2 or 3 doses of COVID-19 vaccines: adenoviral vectored vaccine (ChAdOx1 nCoV-19, Astrazeneca), mRNA vaccine (BNT162b2, Pfizer–BioNTech) or inactivated SARS-COV-2 vaccine (CoronaVac, Sinovac Biotech). IgG antibody against SARS-CoV-2 spike (IgG-S) receptor-binding domain level were quantified at baseline (T1) and 28 days after the first (T2), 2nd (T3) and 3rd (T4) doses by chemiluminescence (SARS-CoV-2-IgG-II Quant-assay, Abbott-Laboratories). Results: 721 patients with IMID were included in the analysis. The median titers of IgG-S (BAU/mL) increased progressively over the times: at baseline was 6.26 (5.41–7.24), T2: 73.01 (61.53–86.62), T3: 200.0 (174.36–229.41) and T4: 904.92 (800.49–1022.97). The multivariate linear regression showed that greater IgG-S titers were associated with pre-exposure to COVID-19 (*p* < 0.001) and BNT162b2 booster vaccine (*p* < 0.001). Rituximab and immunosuppressant drugs were independent factors for low titers (*p* = 0.002, *p* < 0.001, respectively). No serious adverse event was reported. Conclusions: All platforms were safe and induced an increase in IgG-S antibodies. COVID-19 pre-exposure and BNT162b2 booster regimens were predictors of higher humoral immune responses, which is relevant in immunosuppressed populations. Immunosuppressants (mainly rituximab) predicted the lowest antibodies.

## 1. Introduction

Coronavirus disease (COVID-19) has infected more than 770 million people worldwide and caused more than 7 million deaths since December 2019, according to the World Health Organization (WHO) [1]. Vaccination against the severe acute respiratory syndrome Coronavirus 2 (SARS-CoV-2) has proven to be a crucial measure in preventing the widespread consequences of the pandemic.

Patients with immune-mediated inflammatory diseases (IMID) are at increased risk of infections and have been prioritized for urgent vaccination to mitigate COVID-19 risk, consistent with the Rheumatology Society Guidelines on COVID-19 vaccination in patients with immune-mediated rheumatic diseases [2]. The effectiveness of the COVID-19 vaccine in this special population depends on the type and severity of disease, degree of immunosuppression, multimorbidity associated and the vaccine regimens administered [3].

Available data on the effect of biological and synthetic disease-modifying antirheumatic drugs (DMARDs) on vaccine immunogenicity, and vaccination recommendations for individuals with IMID have been summarized and published, as these medications may reduce vaccine responses [4,5,6].

The influence of different vaccine platforms (mRNA-based vaccine, adenoviral vectors or inactivated SARS-CoV-2 vaccines) on the immunogenicity and safety of IMID patients is another point of concern. Some published studies have brought remarkable knowledge related to the efficacy and safety of mRNA-based vaccination in individuals with immune-mediated rheumatic diseases [7,8,9]. However, data on adenovirus-based and inactivated SARS-CoV-2 vaccines in patients with immune-mediated inflammatory diseases are limited [10,11].

Therefore, we conducted a prospective observational multicentric study to evaluate the immunogenicity, effectiveness and safety of different COVID-19 vaccine platforms: inactivated SARS-CoV-2 (CoronaVac, Sinovac Biotech), ChAdOx1 nCoV-19 (AstraZeneca, Oxford) and BNT162b2 (Pfizer-BioNtech) in a large cohort of patients with IMID. Therefore, this study aims to assess the safety and effectiveness of COVID-19 vaccines, as well as the factors influencing vaccine response, by measuring antispike IgG antibodies 28 days after three doses of different COVID-19 vaccine schemes in patients with IMID.

## 2. Materials and Methods

### 2.1. SAFER Study

The “Safety and effectiveness of the COVID-19 Vaccine in Rheumatic Disease (SAFER-Study)” is a Brazilian national, multicentric, observational, longitudinal real-life registry supported by the Brazilian Society of Rheumatology (SBR) of consecutive patients with immune-mediated inflammatory disease who were vaccinated for SARS-CoV-2. The inclusion period was held from June/2021 to March/2024. Follow-up is still ongoing.

SAFER Cohort has included pediatric and adult patients with a prior diagnosis of IMID, according to international consensus. All patients gave written informed consent. It is a non-probability sampling study with enrollment of consecutive patients who met the selection criteria.

All should have completed primary vaccination (2 doses) plus booster dose (3 doses) and attended all 4 follow-up blood collections, from baseline before the first dose to 28 days after all doses. The vaccines available in Brazil during the study were the inactivated SARS-CoV-2 (Sinovac Biotech, Beijing, China), (ChAdOx1 nCoV-19, the AstraZeneca vaccine, Oxford, UK), BNT162b2 (Pfizer-BioNtech, Mainz, Germany) and Ad26.COV2.S (Janssen; Leiden, Netherlands). Patients are being followed up from the inclusion until December of 2024.

**Figure 1:**
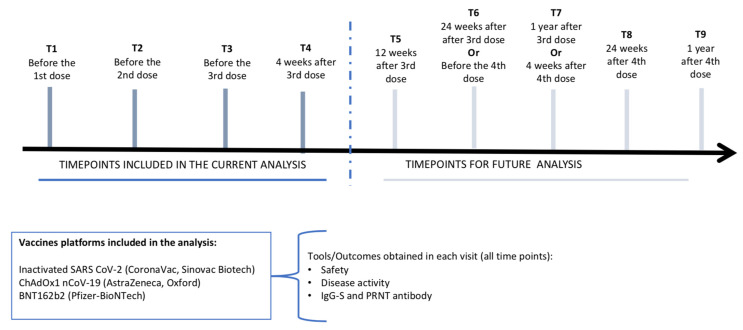
Flowchart of SAFER study with follow-up visits.

The baseline evaluation was conducted prior to the administration of the first dose of the SARS-CoV-2 vaccine. Follow-up visits were scheduled 4 weeks after each vaccine dose and subsequently every 3 months until the 12-month follow-up period (Figure 1).

Additionally, the date, location, vaccine type, vaccination schedule and indication were recorded. Blood samples were taken for immunogenicity analysis. Adverse events, disease flare-ups, and any new immune-mediated manifestations associated with the vaccines were also documented.

### 2.2. Study Population

The study was designed to recruit IMID patients in 10 different states (Espírito Santo, São Paulo, Amazonas, Rio de Janeiro, Goiânia, Paraíba, Ceará, Minas Gerais, Paraná, Rio Grande do Sul) of all 5 Brazilian regions (North, South, Central-West, Southeast, Northeast).

Participants were recruited from university hospital referral centers. This was a real-life study, and all patients had been recommended for vaccination by their attending physicians, who invited and referred them to participate in the study.

### 2.3. Inclusion Criteria

All were adults (≥18 years old) with prior diagnosis of IMID classified according to American College of Rheumatology (ACR) or European League against Rheumatism (EULAR) criteria for rheumatoid arthritis (RA), spondyloarthritis (SpA), systemic lupus erythematosus (SLE), Sjögren’s disease (SjD), inflammatory myopathies (IM), systemic vasculitis (VASC), systemic sclerosis (SSc), mixed connective tissue disease (MCTD) and inflammatory bowel disease (IBD). All should have completed primary vaccination with inactivated vaccine or ChAdOx1 nCoV-19 and plus booster dose (3 doses) with ChAdOx1 nCoV-19 or BNT162b2 platforms and attended all 4 follow-up blood collections, from baseline before the first dose to 28 days after the third dose.

### 2.4. Exclusion Criteria

The exclusion criteria were (1) history of serious adverse events to any vaccine; (2) pregnancy; (3) immunosuppression due to other causes, such as HIV, CD4 < 200 cells/mm^3^, organ transplant, primary immunodeficiency, neoplasia, previous history of thymus diseases (myasthenia gravis, thymoma, cases of absence of thymus or surgical removal); (4) receiving another platform than the inactivated vaccine, ChAdOx1 nCoV-19 or BNT162b2.

Vaccine Postponement Criteria

The vaccines were postponed in situations that could impact the immune response [12,13], as follows. The decision to postpone the vaccination was made by the attending physician. In these cases, patients were only included when they were fit and cleared to begin the planned vaccination. In case the patient should not postpone the vaccination or treatment, they should be excluded from the study.

1. Rituximab in the last 6 months: the vaccine should be postponed for 6 months after the last Rituximab infusion. When possible, wait 2 weeks after the complete vaccination schedule to indicate the next dose of this medication;
2. Blood products transfusion in the last 30 days;
3. Previous suspicion or clinical or laboratory diagnosis (RT-PCR, serology or rapid test) of Sars-Cov-2: physician should wait at least 4 weeks to start vaccination;
4. Plasmapheresis or human immunoglobulin in the previous 30 days;
5. Corticosteroid pulse therapy in the last 30 days.
6. Pulse therapy with cyclophosphamide in the last 30 days;
7. Another inactivated vaccine in the last 14 days;
8. Live attenuated vaccine in the last 28 days.

### 2.5. Vaccines Platforms

We evaluated the three main platforms available to immunosuppressed patients during the pandemic in Brazil. For the primary vaccination it was analyzed ChAdOx1 nCoV-19 (AstraZeneca, Oxford) or inactivated SARS-CoV2 vaccine (CoronaVac, Sinovac Biotech). For the booster third dose we analyzed ChAdOx1 nCoV-19 (AstraZeneca, Oxford, UK) or BNT162b2 (Pfizer-BioNTech).

The CoronaVac COVID-19 vaccine was developed by Sinovac Biotech (China) and consisted of an inactivated whole virus vaccine, derived from the CZ02 coronavirus strain (inactivated SARS-CoV2 vaccine, CoronaVac, Sinovac Biotech). The virus was cultivated in African green monkey kidney cells (Vero Cells), harvested from the supernatant, inactivated with β-propiolactone for 24 h, purified and adsorbed with aluminum hydroxide (Al(OH)_3_) as adjuvant agent [14]. The CoronaVac was authorized for emergency use in Brazil, for individuals with 6 years of age and older on 17 January 2021, by the Agência Nacional de Vigilancia Sanitaria/ANVISA.

The AZD1222 or AstraZeneca COVID-19 vaccine was developed by AstraZeneca, Oxford University (United Kingdom) and was composed of a replication-deficient recombinant chimpanzee adenovirus capsule DNA encoding the SARS-CoV-2 protein spike (ChAdOx1 nCoV-19, AstraZeneca, Oxford). The adenovirus vector consists of unencapsulated icosahedral particles (virions) that contain a single copy of the double-stranded DNA genome. The adenovirus vector enters the target cells through endocytosis, and the released DNA migrates to the cell nucleus, where it is transcribed into mRNA encoding the SARS-CoV2 wild-type spike (S) glycoprotein sequence, with subsequent translation of the spike protein, expressed in a trimeric prefusion conformation that are further processed and presented by immune and non-immune antigen-presenting cells [15]. The Astrazeneca vaccine was authorized for emergency use in Brazil on 12 March 2021, by the Agencia Nacional de Vigilancia Sanitaria/ANVISA.

The Pfizer-BioNTech COVID-19 vaccine was developed by Pfizer/BioNTech (Germany) and comprised an RNA vaccine composed of nucleoside-modified single-stranded, 5′-capped mRNA, generated through cell-free in vitro transcription from a DNA template encoding the SARS-CoV-2-Spike protein (BNT162b2, Pfizer-BioNTech). Upon injection, the mRNA-encapsulated lipid nanoparticles are taken by body cells, delivering the mRNA sequence for translation into viral spike protein that stimulates the immune response. The SARS-CoV-2 membrane-bound spike protein is then recognized by immune cells, eliciting both T-cell and B-cell responses [16].

### 2.6. Ethical Procedures

The study was approved by the National Research Ethics Committee (CONEP) with the study protocol number (CAAE) 43479221.0.1001.5505. It was conducted in accordance with Good Clinical Practice (GCP) guidelines, the International Conference on Harmonization (ICH), the ethical principles established in the Declaration of Helsinki, the Brazilian law 466/2012 and the guidelines of the local ethics committee.

All participants signed the consent form before starting data collection. Participation in the study was voluntary and unpaid. Participants received reimbursement for food and transportation expenses arising from the study, in accordance with local ethical regulations.

Personal identification data was blinded and protected according to international and national regulations to guarantee confidentiality. Only medical researchers had access to patients’ medical records to obtain the data required for the investigation.

### 2.7. Clinical Data

Sociodemographic data, the presence of comorbidities, characteristics and severity of IMID, treatments, clinical characteristics and outcomes of SARS-CoV-2 infection were recorded (previous or during the study).

Patients were classified as having high or low immunosuppression according to the medication (Supplementary Table S1) [12]. High immunosuppression was those with antiproliferative drugs (cyclophosphamide or chlorambucil), mycophenolate mofetil, calcineurin inhibitors (tacrolimus, cyclosporine, sirolimus), azathioprine >2 mg/kg/day, prednisone ≥20 mg/day, methotrexate >20 mg/week or any immunobiological drug.

### 2.8. Safety

Adverse events and disease activity were monitored during the study.

Adverse events (AE) were monitored by 2 strategies:

(1) Diary of adverse events where patient recorded all symptoms up to 28 days after vaccination which included: local reactions (e.g., flushing, pain and edema), general or systemic symptoms (headache; new or worsening myalgia; new or worsening arthralgia; fatigue; nausea, vomiting, diarrhea; pruritus; erythema; chills, fever (≥38°)).

(2) Active surveillance of the occurrence of post-vaccination adverse events by phone contacts, interviews and physical examinations during medical visits before, 1 and 3 months after vaccination. Adverse events were reported according to the type of event; causal relationship (certain, possible, probable, unlikely and unrelated), severity/intensity gradation (1–4 mild-severe/severe), description of the event, duration, start and end date (if applicable), medications, procedures and exams performed, outcome of the event [17]. Patients were asked whether their symptoms had interfered with activities of daily living, missed work or study.

### 2.9. Assessment of Disease Activity

The physician’s global assessment (PGA) of disease activity was recorded at each visit for all patients. PGA < 5 was considered low activity [18]. Further details of disease activity with analysis of other activity indices will be included in new articles with analyses of each disease individually.

### 2.10. Serum Samples

We obtained 10 mL of whole blood samples by a trained professional, from venipuncture of the cubital region in a vacuum tube with a gel separator. After centrifugation (1300× *g* for 15 min, room temperature), the serum aliquots were stored at −80 °C in the Biorepository of SAFER Study until processing.

Blood collection time points were at baseline (T1) and 28 days after the first (T2), 2nd (T3) and 3rd (T4) doses, respectively.

### 2.11. Anti-SARS-CoV-2 Spike Receptor-Binding Domain (RBD) IgG Antibody Quantification

The titers of IgG antibodies to the SARS-CoV-2 spike RBD (IgG-S) were quantified using the SARS-CoV-2 IgG II Quant Assay (Abbott Laboratories, Abbott Park, Chicago, IL, USA), according to the manufacturer’s instructions, using an ARCHITECT i1000SR immunoassay analyzer (Abbott). The results were expressed as binding antibody units (BAU/mL) and seropositivity defined by using 7.1 BAU/mL as the cut-off.

The SARS-CoV-2 IgG II Quant assay is a chemiluminescent microparticle immunoassay (CMIA) designed for the qualitative and semiquantitative detection of IgG antibodies to SARS-CoV-2 in human serum. The assay was used to quantify the serological status of individuals prior to vaccination and also to monitor the antibody response upon COVID-19 vaccination, by quantitatively measuring IgG specific to the spike receptor-binding domain (RBD) of SARS-CoV-2. The assay employed magnetic microparticles coated with RBD recombinant protein.

### 2.12. Statistical Analysis

Humoral response was evaluated four times: at baseline (T1) and 28 days after the first (T2), 2nd (T3) and 3rd (T4) doses. The results were presented as geometric mean IgG titers (GMT) and seroconversion rate. Comparisons between groups and times were performed using the non-parametric Wilcoxon/Mann–Whitney test, using the value of the natural log + 1 of IgG (ln-transformed). In paired comparisons by time, the Bonferroni correction for multiple comparisons was also used.

Subgroup stages were compared by disease, pre-exposure to the vaccine, different types of primary regimens, homologous or heterologous regimens, and whether they received a booster dose with BNT162b2.

A linear regression was performed to investigate the predictors of higher IgG-S titers after booster dose (T4).

Symptomatic cases of COVID-19 at each time point were monitored, according to the vaccines administered and the different diseases.

Safety was assessed by analyzing local and systemic symptoms at each time point and by type of vaccine.

The analyses, tables and graphs were generated using the language R v.4.2 (R Core Team, 2023), the RStudio software (RStudio Team, 2022) and the packages Tidyverse (Wickham, 2019), gt summary (Sjoberg, 2021) and Rstatix (Kassambara, 2023).

## 3. Results

**Figure 2:**
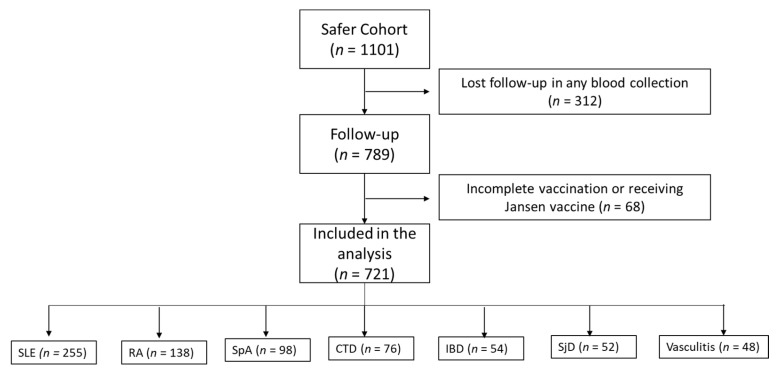
SAFER study population. SLE: systemic lupus erythematosus, RA: rheumatoid arthritis, SpA: spondyloarthritis, CTD: connective tissue disease (inflammatory myopathies, systemic sclerosis, mixed connective tissue disease), IBD: inflammatory bone disease, SjD: Sjögren’s disease.

A total of 1101 participants with IMID were included and followed from the first dose. Three hundred and twelve were excluded because they lost at least one blood collection, and 68 were excluded due to incomplete vaccination or receiving the Janssen vaccine. Seven hundred and twenty-one participants received the complete 3-dose regimen and had blood samples taken during the four study times. Anti-spike IgG antibodies were analyzed in these 721 patients (Figure 2).

The detailed clinical and demographic features of the analyzed patients at inclusion are shown in Table 1. Patients were mostly female (*n* = 562, 77.9%) and self-declared brown skin (*n* = 352, 48.8%), distributed as follows according to the disease: SLE (*n* = 255, 35.4%), RA (*n* = 138, 19.1%), SpA (*n* = 98, 13.6%), SjD (*n* = 52, 7.2%), IBD (*n* = 54, 7.5%), VASC (*n* = 48, 6.7%), other CTD (*n* = 76, 10.5%).

### 3.1. Vaccine Regimens Against COVID-19

Vaccination schedules administered were homologous (25.2% n = 182/721) or heterologous, where the booster dose of a different vaccine platform was used (74.8%, n = 539/721) (Table 2). Serological evidence of exposure to COVID-19 before the first dose was observed in 280 (39%) individuals.

The interval between doses was according to the health minister’s recommendation according to each platform. It means 30 days for inactivated SARS-Cov2 and 60 days to ChAdOx1 nCoV-19. The third booster dose was available approximately 2 months after the second dose. We have observed a mean of 58 (26) days and median (Q1, Q3) 77 days (29 days, 77 days) between the first and second dose. Additionally, for the second and third booster doses, there was a mean (SD) of 76 days (45) and a median (Q1, Q3) of 62 days (56 days, 90 days).

### 3.2. Immunogenicity of the COVID-19 Vaccines

**Figure 3:**
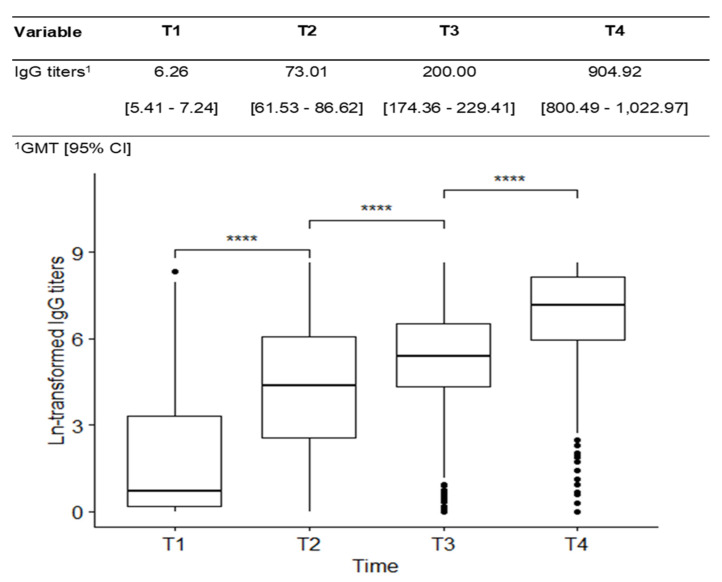
IgG titer comparison in each time along the period was collected in all IMID patients. GMT: geometric mean; T1: at baseline; a T2: 28 days after the first; T3: 28 days after the 2nd; T4: 28 days after the 3rd doses. **** *p* ≤ 0.0001. The results were presented as geometric mean IgG titers (GMT).

All vaccine schedules induced a higher humoral immune response after primary and booster immunization. The median titers of IgG antibodies against SARS-COV-2 increased progressively over the times, at baseline was 6.26 BAU/mL (5.41–7.24), at T2 was 73.01 BAU/mL (61.53–86.62), at T3 was 200.0 BAU/mL (174.36–229.41) and T4 was 904.92 BAU/mL (800.49–1022.97), with a statistical significance difference between all timepoints collected (*p* < 0.001) (Figure 3).

**Figure 4:**
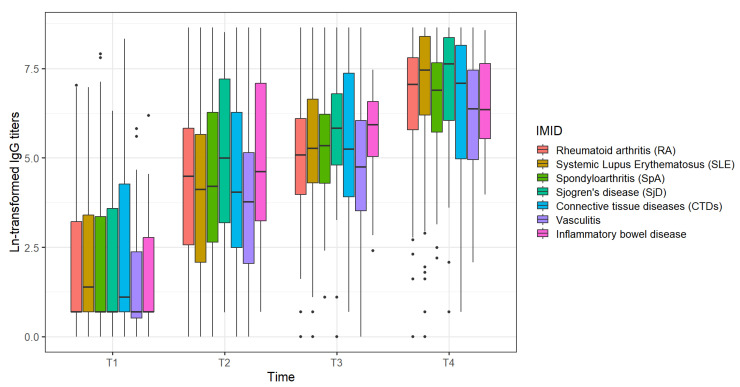
IgG titer comparison by IMID (immune-mediated inflammatory disease) along the study period.

Additionally, stratified analyses and the comparison between IgG titers in each disease group were obtained (Table 3, Figure 4). Vasculitis, inflammatory myositis, systemic sclerosis, inflammatory bowel disease and spondyloarthritis showed a lower humoral immune response compared to Sjögren’s disease and systemic lupus erythematosus (Supplementary Table S2).

**Figure 5:**
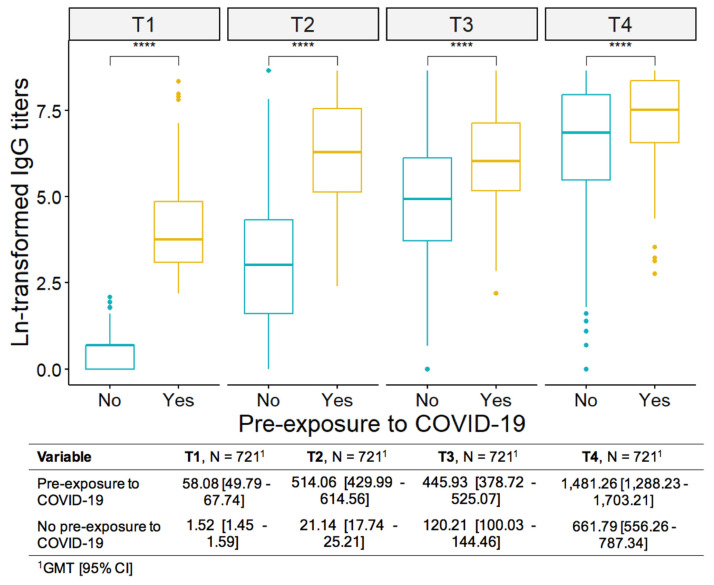
IgG-S antibody titers according to pre-exposure or non-exposure to COVID-19. T1: at baseline; a T2: 28 days after the first; T3: 28 days after the 2nd; T4: 28 days after the 3rd dose. GMT: geometric mean; CI: confidence interval. The results were presented as geometric mean IgG titers (GMT). **** *p* ≤ 0.0001.

At baseline, 39% (*n* = 280/721) had positive IgG-S (>7.1 BAU/mL), indicating previous contact with the SARS-Cov-2 virus (pre-exposed). These individuals had the highest IgG-S antibody titers ever, indicating that the vaccine worked as a booster for the natural response to infection (Figure 5).

Among the homologous regimens, BNT162b2 was more effective than Sinovac and ChAdOx1 nCoV-19 in primary and booster vaccinations (Table 4). Regarding vaccination regimens, the heterologous schemes (*n* = 539; 1199.21 [1048.03–1372.20]) showed greater IgG titers than the homologous alternatives (*n* = 182; 393.06 [309.35–499.42]) (Supplementary Figure S1).

**Figure 6:**
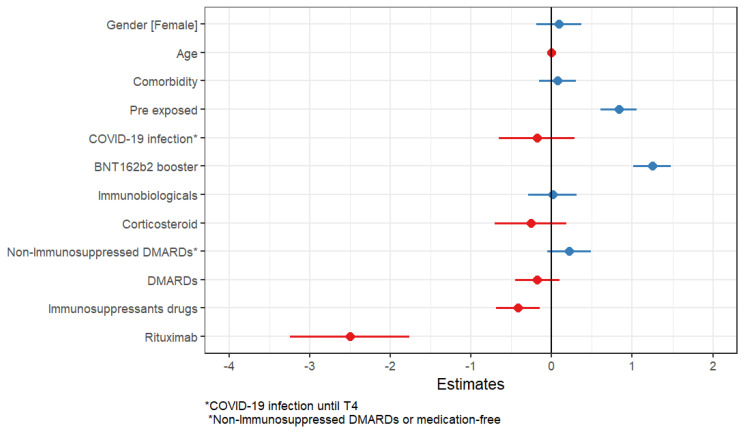
Factors associated with IgG antibody titers after 3rd dose. DMARDs: disease-modifying antirheumatic drug.

Moreover, the multivariate linear regression showed that greater titers of IgG antibodies were also associated with pre-exposure to COVID-19 (*p* < 0.001) and BNT162b2 booster regimens (*p* < 0.001). Pre-exposure to COVID-19 and BNT162b2 booster vaccines were independent predictors of better humoral immune response. Rituximab and immunosuppressant drugs were independent factors for lower titers (*p* = 0.002, *p* < 0.001, respectively). Table 5 and Figure 6.

### 3.3. Safety of the COVID-19 Vaccines

ChAdOx1 nCoV-19 and BNT162b2 vaccines showed more frequency of local adverse events as compared to inactivated SARS-Cov2. Moreover, patients who received ChAdOx1 nCoV-19 vaccine had more systemic reactions than those who received inactivated SARS-Cov2 and BNT162b2. Regarding vaccine safety and risk of adverse events, all vaccines were safe, and no serious adverse events were observed during the follow-up. The booster doses of all vaccination schedules had a similar frequency of local and systemic adverse effects in all vaccine platforms. The incidence of each adverse event after the primary regimens and booster dose is detailed in Table 6 and Table 7.

## 4. Discussion

This study plays a relevant role in current scientific research as it provides important results on the safety and immunogenicity of different COVID-19 vaccination schedules in a population of patients with immune-mediated inflammatory diseases during the SARS-CoV-2 pandemic. There are some challenges faced by the IMID population, such as reduced vaccine efficacy, potential unresponsiveness to vaccination, and increased risk of severe outcomes or life-threatening complications.

All vaccines induced a higher humoral immune response after primary and booster immunization. Booster doses have been proven to be safe and effective in increasing SARS-CoV-2-specific antibody titers, which is particularly crucial for protecting immunocompromised individuals who are at the highest risk of severe COVID-19 [19]. Our findings emphasize the advantages of a vaccine booster in patients with IMID, as it ensures a high seroconversion rate and produces a threefold increase in the quantitative humoral response compared to the initial vaccination. In a recently published subanalysis of this population, there was evidence that increasing the number of vaccine doses was a protective factor for hospitalization for COVID-19 (OR: 0.37; CI: 0.15–0.9; *p* = 0.032) [20].

We observed that the BNT162b2 booster was a predictor of better humoral immune response. Several studies have demonstrated this effect. Farroni et al. showed that a booster dose of the BNT162b2 vaccine significantly increased antibody titers in patients with rheumatoid arthritis, in which almost all patients with RA (51/52, 98.1%) had a positive anti-RBD-IgG response 4–6 weeks after the third dose [21]. Furthermore, Bieber et al. reported that the BNT162b2 booster was linked to improved COVID-19 outcomes in patients with autoimmune rheumatic diseases, reflecting a strong humoral response. The hazard ratio for SARS-CoV-2 infection in the primary vaccination group was 0.143 (95% CI: 0.095 to 0.214, *p* < 0.001), while in the booster group, it was 0.017 (95% CI: 0.009 to 0.035, *p* < 0.001) [22]. Additionally, an epidemiological study demonstrated increased efficacy of mRNA vaccines in preventing COVID-19-related hospitalizations from the second to the third dose, with efficacy rising from 82% to 97% in immunocompetent individuals and from 69% to 88% in immunocompromised individuals [23]. Over the observation period (28 days after the third dose), there were 168 cases of SARS-Cov-2 infection, confirmed by RT-PCR, and probably other asymptomatic cases, but this variable was not controlled by the study [20].

Immunosuppressants drugs and biological therapy with rituximab were predictors of lower IgG titers. Immunosuppressive drugs and biological therapy with rituximab were identified as predictors of lower IgG titers. Both prospective studies and meta-analyses have shown that rituximab and B-cell-depleting therapies are associated with seroconversion rates of less than 70% [9,24,25]. In a meta-analysis involving 18 cohorts. In a meta-analysis of 18 cohorts, the seroconversion rate for patients receiving rituximab was significantly lower, at 29.6% (95% CI: 13.8–52.4, I² = 37%) [25]. Similarly, our findings align with previous research indicating that patients treated with B-lymphocyte-depleting agents, such as rituximab, and lymphocyte proliferation inhibitors, like mycophenolate (including mycophenolate mofetil and mycophenolic acid), demonstrate suboptimal antibody responses after receiving two doses of the COVID-19 vaccine [24]. The geometric mean of anti-S antibody titers was 1371 (CI: 809–2323) in patients using antimetabolites (methotrexate, mycophenolate, azathioprine, leflunomide, teriflunomide or 6-mercaptopurine), compared to 1985 (CI: 1293–3047) in those not using these medications, following two doses of mRNA-based SARS-CoV-2 vaccination [24].

We also evaluated the immune responses in different diseases. Vasculitis, inflammatory myositis, systemic sclerosis, inflammatory bowel disease and spondyloarthritis showed lower humoral immune responses compared to Sjögren’s disease and systemic lupus erythematosus. It may be related to the degree of immunosuppression and the degree of activity of each patient during follow-up. Analysis of factors that impacted the immunogenicity of COVID-19 vaccines in each disease group is ongoing and will be shown in future publications to further evaluate the vaccination effectiveness in each population.

Vaccine safety was also studied [8,10,11]. The three COVID-19 vaccines (ChAdOx1 nCoV-19, BNT162b2, inactivated SARS-Cov2) have shown some common local and systemic reactions, such as fever, chills, fatigue, headache and muscle pain, that have frequently been seen after vaccinations. No serious adverse events were reported. ChAdOx1 nCoV-19 showed a higher frequency of local and systemic adverse events. The higher frequency of local and systemic adverse events associated with the ChAdOx1 nCoV-19 COVID-19 vaccine (ChAdOx1 nCoV-19) has been well-documented in several studies [11,26,27]. In a cohort with health workers, ChAdOx1 nCoV-19 showed more symptoms than Sinovac in both doses (87% versus 61%, *p* < 0.001 after the first dose; 57% versus 43%, *p* < 0.001, after 2nd dose), respectively [26]. Our study supported the above findings and showed that the reported side effects were mild in intensity and did not require hospitalization.

When we designed the study, our goal was to include all platforms distributed by the Brazilian public health system. However, when we began the analysis, we found that only a few individuals had received the Janssen vaccine. To ensure a more robust statistical analysis, we decided to focus on the three main platforms available to immunosuppressed patients during the pandemic in Brazil. Consequently, the platforms and combinations analyzed were those that were more commonly used in real-life situations in Brazil.

This study has some limitations. The main limitation was the difficulty patients had in adhering to and returning for visits, especially on days when vaccines were not administered. Patients underwent a large number of blood sample collections at each visit, and many had compromised venous access. Patients also had transportation difficulties, lived in other cities and many were hesitant to leave the house because they were socially isolated. These factors impacted adherence and led to losses in follow-up.

This study, being a real-life analysis rather than a prospectively hyper-controlled experimental setting, includes a significant number of variables, such as geographical locations, age, various diseases and treatments, different vaccination platforms, as well as exceptions and delays. We made an effort to address the confounding factors using multiple regression analysis. While the majority of these variables have been analyzed, not all have been included. For instance, we did not account for variables such as ethnicity, the transmissibility rate in each geographic area, and the distribution of vaccine platforms per center. We consider these minor variables; however, they could still influence the results.

The SAFER study evaluated IMID patients from baseline until 1 year after the 4th dose in nine follow-up time points. In this manuscript, we analyzed the primary vaccination (first and second dose) and 4 weeks after the third booster dose. Future analysis will show the follow-up, including bivalent analysis.

## 5. Conclusions

In summary, our study is of great importance in this scenario, as we present a large and diverse cohort of immune-mediated inflammatory diseases and evaluate different vaccine platforms in these patients. All vaccine platforms against COVID-19, in a complete vaccination schedule with three doses, were safe and induced an increase in humoral immune response, which could improve protection against COVID-19 in IMID patients. COVID-19 pre-exposure and BNT162b2 booster regimens were predictors of higher humoral immune responses, which is relevant in this immunosuppressed population, regardless of age, sex and comorbidities. Rituximab and immunosuppressant drugs (mainly rituximab) predicted the worst immune response. The results are relevant to planning strategies for COVID-19 immunization in autoimmune disease patients.

## Figures and Tables

**Table 1:** Demographic characteristics of the SAFER study participants at the baseline.

Variables	*n* = 721
Age (years)-median (IQR)	41 (32–51)
Gender	*n* (%)
Female	562 (77.9)
Male	159 (22.1)
Self-declared skin color	*n* (%)
Brown	352 (48.8)
White	267 (37)
Black	91 (12.6)
Asian	8 (1.1)
Indigenous	3 (0.4)
BMI (Kg/m²)-median (IQR)	26 (23–30)
Comorbidities	*n* (%)
Systemic arterial hypertension	169 (23.4)
Diabetes mellitus	40 (5.5)
Heart disease	30 (4.2)
Chronic renal disease	7 (1)
Smoking	39 (5.4)
Immune-mediated inflammatory disease	*n* (%)
Systemic lupus erythematosus (SLE)	255 (35.4)
Rheumatoid arthritis (RA)	138 (19.1)
Spondyloarthritis (SpA)	98 (13.6)
Connective tissue diseases (CTDs)	76 (10.5)
Inflammatory bowel disease (IBD)	54 (7.5)
Sjögren’s disease (SjD)	52 (7.2)
Vasculitis (VASC)	48 (6.7)
Disease activity ^1^	*n* (%)
Moderate/High Activity	156/630 (25)
No/low Activity	474/630 (75)
Drugs use	*n* (%)
Non-Immunosuppressed DMARD or drug-free	375/721 (52)
Corticosteroid	48/721 (6.7)
DMARDs	207/721 (29)
Immunosuppressed drugs	205/721 (28)
Immunobiologicals	195/721 (27)
Rituximab	16/721 (2.2)
COVID-19 exposure before first dose ^2^	280 (38.8)

BMI: Body mass index; IQR: interquartile; DMARDs: disease-modifying antirheumatic drugs. 1 Disease activity according to physician global assessment (PGA). 2 Pre-exposure: IgG ≥ 7.1 BAU/mL on T1.

**Table 2:** Vaccination schedule in primary and booster immunization.

Characteristic	*N* = 721
Primary Vaccination	
ChAdOx1 nCoV-19	369/721 (51%)
Inactivated SARS-CoV2	318/721 (44%)
BNT162b2	34/721 (4.7%)
Booster dose	
BNT162b2	496/721 (69%)
ChAdOx1 nCoV-19	217/721 (30%)
Inactivated SARS-CoV2	8/721 (1%)
Schedule type	
Heterologous	539/721 (75%)
Homologous	182/721 (25%)
BNT162b2 Booster	496/721 (69%)
Vaccination schedule	
Inactivated + Inactivated + BNT162b2	233/721 (32%)
ChAdOx1 nCoV-19 + ChAdOx1 nCoV-19 + BNT162b2	229/721 (32%)
ChAdOx1 nCoV-19 + ChAdOx1 nCoV-19 + ChAdOx1 nCoV-19	140/721 (19%)
Inactivated + Inactivated + ChAdOx1 nCoV-19	77/721 (11%)
BNT162b2 + BNT162b2 + BNT162b2	34/721 (4.7%)
Inactivated + Inactivated + Inactivated	8/721 (1.1%)

**Table 3:** IgG titer comparison in each disease along the period collected.

Characteristic	Rheumatoid Arthritis (RA), *n* = 138 ^1^	Systemic Lupus Erythematosus (SLE), *n* = 255 ^1^	Spondyloarthritis (SpA), *n* = 98 ^1^	Sjögren’s Disease (SjD), *n* = 52 ^1^	Connective Tissue Diseases (CTDs), *n* = 76 ^1^	Vasculitis, *n* = 48 ^1^	Inflammatory Bowel Disease, *n* = 54 ^1^
IgG-T1	6.45 [4.59–9.07]	6.87 [5.47–8.61]	5.94 [3.82–9.25]	6.09 [3.49–10.62]	8.60 [5.05–14.66]	3.78 [2.34–6.10]	4.26 [2.78–6.51]
IgG-T2	75.22 [52.86–107.05]	56.49 [42.27–75.50]	85.87 [54.11–136.26]	183.55 [96.80–348.02]	63.62 [35.34–114.50]	45.55 [23.18–89.51]	128.54 [73.17–225.82]
IgG-T3	151.73 [116.46–197.70]	203.83 [158.74–261.74]	218.49 [163.84–291.36]	321.01 [192.95–534.07]	188.05 [109.15–323.97]	140.20 [76.36–257.39]	299.23 [221.91–403.47]
IgG-T4	799.14 [602.13–1060.61]	1172.58 [955.35–1439.20]	889.86 [683.68–1158.23]	1274.68 [822.77–1974.80]	668.46 [413.89–1079.63]	537.15 [321.79–896.65]	660.05 [476.10–915.08]

^1^ GMT [95% CI]. T1: at baseline; a T2: 28 days after the first; T3: 28 days after the 2nd; T4: 28 days after the 3rd dose. GMT: geometric mean; CI: confidence interval. The results were presented as geometric mean IgG titers (GMT).

**Table 4:** Comparison of humoral immune response (IgG-S in BAU/mL) among different vaccine platforms and schemes.

Primary	Inactivated SARS-CoV2, *N* = 318 ^1^	ChAdOx1 nCoV-19, *N* = 369 ^1^	BNT162b2, *N* = 34 ^1^			
T2	42.39 [34.19–52.57]	112.63 [87.20–145.48]	106.62 [42.00–270.63]			
T3	95.96 [81.29–113.28]	312.06 [256.32–379.93]	1538.95 [957.34–2473.9]			
Booster Schedules	Inactivated + Inactivated + Inactivated *N* = 8 ^1^	Inactivated + Inactivated + ChAdOx1 nCoV-19 *N* = 77 ^1^	Inactivated + Inactivated +BNT162b2 *N* = 233 ^1^	ChAdOx1 nCoV-19 + ChAdOx1 nCoV-19 + ChAdOx1 nCoV-19 *N* = 140 ^1^	ChAdOx1 nCoV-19 + ChAdOx1 nCoV-19 + BNT162b2 *N* = 229 ^1^	BNT162b2 + BNT162b2 + BNT162b2 *N* = 35 ^1^
T4	160.06 [50.46–507.69]	895.32 [683.83–1172.22]	1165.37 [939.39–1445.70]	287.69 [224.58–368.54]	1372.77 [1117.13–1686.92]	1795.18 [1158.49–2781.79]

^1^ GMT [95% CI]. T1: at baseline; a T2: 28 days after the first; T3: 28 days after the 2nd; T4: 28 days after the 3rd dose. GMT: geometric mean; CI: confidence interval. The results were presented as geometric mean IgG titers (GMT).

**Table 5:** Multiple regression analysis considering variables that could influence the immunogenicity results based on IgG antibody titers after 3rd dose.

Characteristic	95% CI ^1^	*p*-Value
Gender		
Male	-	-
Female	−0.02–0.36	0.6
Age	−0.01–0.01	0.4
Comorbidity	−0.16–0.30	0.5
Pre-exposed	0.62–1.1	<0.001
COVID-19 infection until T4	−0.69–0.26	0.4
BNT162b2 booster	1.0–1.5	<0.001
Immunobiologicals	−0.28–0.32	0.9
Corticosteroid	−0.70–0.19	0.3
Non-Immunosuppressed DMARDs or medication-free	−0.04–0.51	0.10
DMARDs	−0.44–0.11	0.2
Immunosuppressants drugs	−0.70–- 0.16	0.002
Rituximab	−3.3–- 1.8	<0.001

^1^ CI = Confidence Interval.

**Table 6:** Occurrence of adverse events following vaccination by each primary schedule.

Characteristic	Inactivated, N = 318 ^1^	ChAdOx1 nCoV-19, N = 369 ^1^	BNT162b2, N = 34 ^1^	*p*-Value ^2^
Local reactions	174/315 (55%)	319/369 (86%)	26/34 (76%)	<0.001
Erythema	37/315 (12%)	86/369 (23%)	5/34 (15%)	<0.001
Ecchymosis	23/315 (7.3%)	68/369 (18%)	3/34 (8.8%)	<0.001
Lesion	35/315 (11%)	60/369 (16%)	6/34 (18%)	0.12
Itching	17/35 (49%)	28/60 (47%)	4/6 (67%)	0.7
Swelling	53/315 (17%)	143/369 (39%)	12/34 (35%)	<0.001
Induration	55/315 (17%)	130/369 (35%)	11/34 (32%)	<0.001
Local pain	159/315 (50%)	308/369 (83%)	24/34 (71%)	<0.001
Systemic reactions	188/315 (60%)	305/369 (83%)	20/34 (59%)	<0.001
Nausea/Vomiting	71/315 (23%)	123/369 (33%)	5/34 (15%)	0.002
Tiredness	97/315 (31%)	201/369 (54%)	12/34 (35%)	<0.001
Headache	129/315 (41%)	221/369 (60%)	16/34 (47%)	<0.001
Muscle pain	106/315 (34%)	196/369 (53%)	15/34 (44%)	<0.001
Joint pain	109/315 (35%)	210/369 (57%)	14/34 (41%)	<0.001
Fever	45/315 (14%)	127/369 (34%)	5/34 (15%)	<0.001
Dizziness	72/315 (23%)	108/369 (29%)	7/34 (21%)	0.12
Other complaints	74/315 (23%)	143/369 (39%)	11/34 (32%)	<0.001

^1^ n/N (%); ^2^ Pearson’s Chi-squared test.

**Table 7:** Occurrence of Adverse events following booster vaccines.

Characteristic	Inactivated, N = 8 ^1^	ChAdOx1 nCoV-19, N = 217 ^1^	BNT162b2, N = 496 ^1^	*p*-Value ^2^
Local reactions	3/4 (75%)	124/204 (61%)	280/413 (68%)	0.2
Systemic reactions	3/4 (75%)	115/204 (56%)	216/413 (52%)	0.4

^1^ n/N (%); ^2^ Fisher’s exact test.

## Data Availability

The data are not publicly available due to privacy restrictions and are only available upon request to the corresponding author at val.valim@gmail.com.

## Supplementary Materials

The following supporting information can be downloaded at: https://www.mdpi.com/article/10.3390/vaccines12121367/s1, Figure S1: Comparison of humoral immune response (IgG-S in BAU/mL) among homologous and heterologous scheme vaccinations; Table S1: Immunosuppression degree conferred by drugs used to treat immune-mediated diseases according to Brazilian societies ^a^ (BRS/BDS/GEDIIB). Table S2: Analysis of comparison of humoral immune response (IgG-S in BAU/mL) among pairs of diseases at each time point.
