# Supplementary material for: Immunogenicity and Safety According to Immunosuppressive Drugs and Different COVID-19 Vaccine Platforms in Immune-Mediated Disease: Data from SAFER Cohort
Source: Vaccines (Basel). 2024 Dec 3;12(12):1367. doi: 10.3390/vaccines12121367 (PMC11680192; doi:10.3390/vaccines12121367)
Supplement: Supplementary file 1 [file vaccines-12-01367-s001.zip › vaccines-3313580-supplementary.pdf]

Supplementary Table S1. Immunosuppression degree conferred by drugs used to treat immune-mediated diseases according to Brazilian societies [a] (BRS/BDS/GEDIIB).

Non-immunosuppressed
No drug treatment<br>Only using sulfasalazine or hydroxychloroquine or mesalazine or acitretin<br>Using topical, inhaled, peri or intra-articular corticosteroids
Low immunosuppression degree<br>Methotrexate at a dose of ≤0.4 mg/kg/week or ≤20 mg/week<br>Leflunomide at a dose of ≤20 mg/day<br>Corticosteroid at a dose of ≤20 mg/day (or 2 mg/kg/day for weigh <10 kg) prednisone or equivalent
High immunosuppression degree<br>Corticosteroid at a dose of ≥20 mg/day (or >2 mg/kg/day for patients weighing <10 kg) prednisone or equivalent, for a period ≥14 days<br>Pulse Therapy with methylprednisolone<br>Immunosuppressants as mycophenolate mofetil or sodic, cyclosporine, cyclophosphamide tacrolimus, azathioprine<br>JAK inhibitors, such as tofacitinib<br>b-DMARD [b]

[a] Brazilian Rheumatology Society (SBR), Brazilian Dermatology Society (SBD) and the Brazilian Inflammatory Bowel Diseases Study Group (GEDIIB).
[b] bDMARD: biologic disease modifying antirheumatic drugs.

Supplementary Table S2: Analysis of comparison of humoral immune response (IgG-S in BAU/mL) among pairs of diseases at each time point.

Group 1	Group 2	N1	N2	Statistic T1	p-value T1	Statistic T2	p-value T2	Statistic T3	p-value T3	Statistic T4	p-value T4
Rheumatoid arthritis (RA)	Systemic Lupus Erythematosus (SLE)	138	255	16,308.0	0.231	18,899.0	0.225	15,645.5	0.07	14,777.0	**0.009***
Rheumatoid arthritis (RA)	Spondyloarthritis(SpA)	138	98	7,116.5	0.492	6,563.0	0.701	5,995.0	0.138	6,865.0	0.843
Rheumatoid arthritis (RA)	Sjogren's syndrome (SS)	138	52	3,747.0	0.638	2,758.0	**0.014***	2,419.0	**< 0.001***	2,896.0	**0.04***
Rheumatoid arthritis (RA)	Connective tissue diseases (CTDs)	138	76	4,861.5	0.377	5,467.0	0.608	4,667.5	0.184	5,179.5	0.883
Rheumatoid arthritis (RA)	Vasculitis	138	48	3,803.5	0.125	3,753.5	0.17	3,373.5	0.849	3,764.5	0.159
Rheumatoid arthritis (RA)	Inflammatory bowel disease	138	54	4,155.5	0.214	3,227.0	0.15	2,653.0	**0.002***	4,254.0	0.127

Systemic Lupus Erythematosus (SLE)	Spondyloarthritis(SpA)	255	98	14,043.5	0.071	11,327.0	0.174	12,619.0	0.886	14,670.0	**0.011***
Systemic Lupus Erythematosus (SLE)	Sjogren's syndrome (SS)	255	52	7,269.0	0.273	4,811.5	**0.002***	5,533.0	0.06	6,474.0	0.789
Systemic Lupus Erythematosus (SLE)	Connective tissue diseases (CTDs)	255	76	9,623.0	0.928	9,511.5	0.808	9,644.0	0.95	11,040.5	0.064
Systemic Lupus Erythematosus (SLE)	Vasculitis	255	48	7,521.0	**0.012***	6,519.0	0.474	6,816.5	0.211	7,758.0	**0.003***
Systemic Lupus Erythematosus (SLE)	Inflammatory bowel disease	255	54	8,223.5	**0.025***	5,624.0	**0.035***	6,133.0	0.208	9,114.0	**< 0.001***
Spondyloarthritis(SpA)	Sjogren's syndrome (SS)	98	52	2,495.0	0.835	2,059.0	0.054	1,987.0	**0.027***	2,020.0	**0.037***
Spondyloarthritis(SpA)	Connective tissue diseases (CTDs)	98	76	3,266.0	0.163	4,021.5	0.367	3,638.0	0.795	3,670.0	0.871
Spondyloarthritis(SpA)	Vasculitis	98	48	2,540.5	0.43	2,699.0	0.149	2,623.5	0.259	2,672.0	0.183
Spondyloarthritis(SpA)	Inflammatory bowel disease	98	54	2,793.0	0.57	2,373.0	0.294	2,235.0	0.114	3,064.0	0.108
Sjogren's syndrome (SS)	Connective tissue diseases (CTDs)	52	76	1,742.0	0.256	2,452.0	**0.021***	2,214.0	0.249	2,289.0	0.129
Sjogren's syndrome (SS)	Vasculitis	52	48	1,387.5	0.335	1,666.5	**0.004***	1,596.0	**0.016***	1,619.0	**0.01***
Sjogren's syndrome (SS)	Inflammatory bowel disease	52	54	1,519.0	0.467	1,575.0	0.281	1,550.0	0.358	1,885.0	**0.002***
Connective tissue diseases (CTDs)	Vasculitis	76	48	2,196.5	0.056	1,954.0	0.506	2,011.0	0.339	2,007.5	0.347

Connective tissue diseases (CTDs)	Inflammatory bowel disease	76	54	2,437.5	0.068	1,703.0	0.1	1,905.0	0.489	2,364.0	0.141
Vasculitis	Inflammatory bowel disease	48	54	1,240.5	0.71	996.0	**0.045***	966.0	**0.027***	1,268.0	0.854

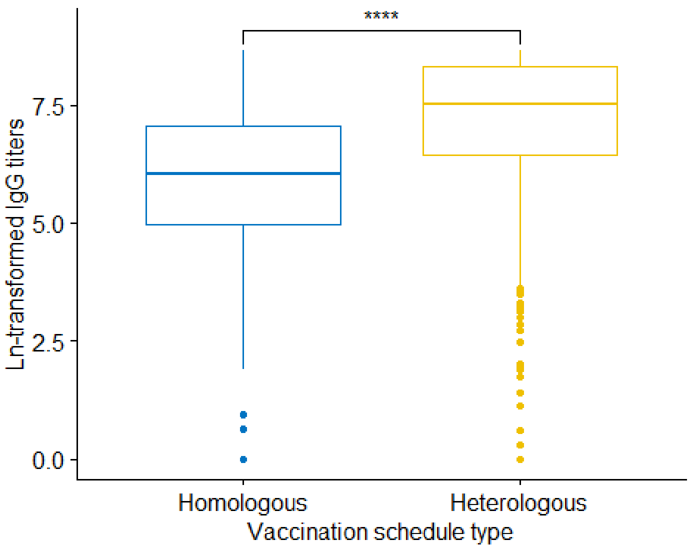

Supplementary Figure S1. Comparison of humoral immune response (IgG-S in BAU/mL) among homologous and heterologous scheme vaccinations.
